# Nicotine delivery, tolerability and reduction of smoking urge in smokers following short-term use of one brand of electronic cigarettes

**DOI:** 10.1186/s12889-015-2349-2

**Published:** 2015-09-30

**Authors:** Carl D. D’Ruiz, Donald W. Graff, X. Sherwin Yan

**Affiliations:** ITG Brands, LLC, A.W. Spears Research Center, R&D, Department of Scientific Affairs, 420 N. English Street, P.O. Box 21688, Greensboro, NC 27420-1688 USA; Celerion, Lincoln, NE USA; Lorillard Tobacco Company, R&D, Department of Scientific Affairs, Greensboro, NC USA

**Keywords:** Electronic cigarettes, Clinical trial, Pharmacokinetics, Pharmacodynamics, Smoking urge, Tolerability, Adverse events, Plasma nicotine, Crossover study

## Abstract

**Background:**

This randomized, partially single-blinded, 6-period crossover clinical study of adult smokers compared the nicotine pharmacokinetics, impacts on smoking urge and tolerability of various formulations of one brand of e-cigarettes with that of a tobacco cigarette.

**Methods:**

Five e-cigarettes with different e-liquid formulations containing 1.6 % and 2.4 % nicotine and a conventional tobacco cigarette were randomized among 24 subjects under two exposure sessions consisting of a 30-min controlled and a one-hour *ad lib* use period to assess plasma nicotine levels, impacts on smoking urge and adverse events. The 30-min controlled use session comprised an intensive use of the e-cigarettes with a total of 50 puffs taken every 30 s for comparison to a single conventional cigarette having a typical machine-measured nicotine yield (~0.8 mg). *Ad lib* product use conditions provided insight into more naturalistic product use behaviors and their accompanying smoking urge reductions. Adverse events (AEs) were assessed by the Principal Investigator.

**Results:**

Significant (*p* < 0.05) increases in plasma nicotine concentrations occurred within 10 min of controlled e-cigarette use and significant (*p* < 0.001) reductions from baseline smoking urge were observed within 5 min. E-cigarette and cigarette nicotine plasma levels were comparable for up to one hour of use. After both sessions (90 min), nicotine exposure was the highest for the cigarette, with all e-cigarettes showing 23 % to 53 % lower plasma concentrations. During controlled use, peak reduction in smoking urge for e-cigs occurred later than for the cigarette. After completion of both sessions, significant smoking urge reduction persisted for most of the tested e-cigarettes, albeit at levels lower than that provided by the tobacco cigarette. Nicotine content, vehicle differences, and the presence of menthol did not significantly affect smoking urge reduction by the e-cigarettes. No subjects were discontinued due to AEs. The most frequently reported AEs events included cough, throat irritation, headache, and dizziness.

**Conclusions:**

Blood plasma nicotine levels obtained from short-term use of e-cigarettes containing 1.6 % and 2.4 % nicotine were significant, but lower than those of conventional tobacco cigarettes, yet the reduction in craving symptoms were broadly comparable. The types of AEs were consistent with other research studies of longer duration that have reported that use of e-cigarettes by adult smokers is well-tolerated.

**Trial Registration:**

http://ClinicalTrials.gov identifier: NCT02210754. Registered 8 August 2014.

## Background

Smoking remains the leading preventable cause of premature death in the United States [1]. Electronic cigarettes (e-cigarettes) are rapidly becoming a popular alternative to cigarette smoking worldwide and are garnering significant attention as potentially reduced-exposure replacements for conventional cigarettes (cigarettes) [2–6]. E-cigarettes consist of a battery, heating component, and a cartridge containing tobacco-derived nicotine in a solution composed of glycerin and/or propylene glycol (PG), and flavorings. Upon activation, the heating element heats the solution to generate an aerosol that is inhaled by the consumer in a manner that mimics smoking.

Pharmacokinetic studies with early-generation e-cigarettes found that they delivered markedly lower levels of plasma nicotine than conventional cigarettes [7, 8]. More recent studies have reported that experienced subjects using later-generation e-cigarettes containing 9 to 24 mg/mL of nicotine attain significant increases in plasma nicotine concentrations over baseline values that can be similar to those obtained from conventional cigarette smoking [9–11]. These results suggest that an acclimation to the product may be necessary for naïve smokers to become familiar with the characteristics of the specific e-cigarettes in order to effectively use them in a subjectively enjoyable manner. Moreover, these studies also demonstrated a reduction in smoking urge or abstinence symptoms following e-cigarette use, even for products that deliver less nicotine than conventional tobacco cigarettes. This is quite consistent with a perspective that the substantial sensory and behavioral aspects of cigarette smoking that are mimicked by e-cigarettes may provide meaningful relief of the cigarette cravings commonly reported by abstinent smokers.

Surveys and clinical studies evaluating the impacts, tolerability and adverse events (AEs) associated with e-cigarette use suggest that they are generally well-tolerated following short-term use [1, 9, 12–16]. Commonly reported AEs include symptoms such as mouth and throat irritation, light-headedness, dizziness and dry cough.

The primary objective of this study was to examine the nicotine blood plasma levels and smoking urge impacts of various formulations of one brand of e-cigarette with that of a tobacco cigarette. The tested products contained different flavorings, aerosol forming excipients (i.e., propylene glycol, glycerin) and nicotine levels. The secondary objectives were to assess the tolerability and any adverse events associated with the study products following short-term use under intensive controlled use conditions, as well as under more natural *ad-lib* use conditions.

## Methods

### Participants

The study protocol and the informed consent forms were approved by Chesapeake IRB, Columbia, MD. A total of 107 potential subjects were recruited from the Lincoln, NE area using standard advertising methods (i.e., print and radio advertisements) and from a database of subjects who had previously participated a clinical research study or who had expressed interest in participating in a study. All potential subjects were provided details regarding the study and written informed consent was obtained prior to completion of any study procedures. Sixty-one subjects were excluded for not meeting the predefined inclusion/exclusion criteria, while five subjects declined to participate prior to enrollment and three subjects were excluded because the study had reached the recruitment target of 38 eligible subjects. The 38 subjects meeting the eligibility criteria participated in a 7-day at-home lead-in period during which they used and became familiar with two of e-cigarette study products (non-menthol and menthol, 2.4 % nicotine with glycerin). All subjects who participated in the at-home lead-in period were allowed to check in at the start of clinical conduct. Two subjects chose not to participate further after the lead-in period and one subject failed further screening requirements at check-in. A final study population of 24 subjects were enrolled into the study and randomized into one of six product usage sequences with four subjects per sequence. One subject withdrew consent after completion of the first product use on Day 1 of the trial due to a personal reason (family emergency). All subjects participating in the study from the time of the lead-in period were paid for their participation.

The main criteria for inclusion were as follows: healthy adult male and female smokers, 21 to 65 years of age, inclusive; smoker for at least 12 months and currently smoked an average of 10 or more manufactured cigarettes per day (no restriction on brand-style); positive urine cotinine at screening (≥ 500 ng/mL); and exhaled carbon monoxide CO > 10 ppm at screening. Exclusion criteria included: history or presence of clinically significant mental or physical health conditions; females who were pregnant or breastfeeding; high blood pressure; body mass index < 18 kg/m^2^ or > 40 kg/m^2^; acute illnesses (e.g., upper respiratory infection, viral infection) requiring treatment within 2 weeks prior to check-in; use of prescription smoking cessation treatments, anti-diabetic or insulin drugs or medications known to interact with cytochrome P450 2A6; positive urine screen for alcohol or drugs of abuse; and self-reported puffers (i.e., smokers who draw smoke from the cigarette into the mouth and throat but do not inhale). Subjects who had used any tobacco- or nicotine-containing products other than manufactured cigarettes or e-cigarettes within 28 days of in-clinic product use were also excluded.

### Products tested

A rechargeable version of an e-cigarette that is currently sold in retail outlets throughout the United States was used in this study. The rechargeable e-cigarette consists of a battery segment and a cartomizer segment comprising the heating unit and a liquid reservoir which can be separated from the battery for recharging or replacement when the e-liquid is depleted. The battery operates at a voltage of 3.7 volts (nominal) and the resistance of the heating element is approximately 3 ohms. The maximum operating temperature is dependent on both the state of reservoir fluid fill and on the manner of use and was not recorded in this study.

Two commercial e-cigarette products that contained 16 mg/mL (1.6 %) USP grade nicotine were used in this study. As well, three non-commercial products that contained 24 mg/mL (2.4 %) USP grade nicotine were used in this study to evaluate various product characteristics considered important to further product development. In addition to nicotine, the e-cigarettes used in this study contained USP grade glycerin and/or propylene glycol (as described below), distilled water (<20 %), citric acid (<1.0 %) and natural or artificial flavors (<10 %). The nicotine yield of the conventional cigarette used in the study was approximately 0.8 mg per cigarette [17]. The study products included:

- **Product A:** Classic Tobacco flavored e-cigarette (2.4 % nicotine, ~75 % glycerin)
- **Product B:** Classic Tobacco flavored e-cigarette (2.4 % nicotine, ~50 % glycerin/~20 % propylene glycol)
- **Product C:** Menthol flavored e-cigarette (2.4 % nicotine, ~75 % glycerin)
- **Product D:** Classic Tobacco flavored e-cigarette (1.6 % nicotine, ~75 % glycerin)
- **Product E:** Classic Tobacco flavored e-cigarette (1.6 % nicotine, ~50 % glycerin/~20 % propylene glycol)
- **Product F:** Tobacco Cigarette

### Study design

This was a randomized, partially single-blinded, six-period crossover study conducted at a single independent research center (Celerion, Lincoln, NE). Twenty-four subjects were randomly selected from the pool of 38 subjects who participated in the at-home lead-in period were enrolled into the testing phase of the trial and randomized to assigned product sequences. The subjects checked into the clinic on Day −2 and abstained from using of nicotine-containing products until product use on Day 1. Days 1, 3, 5, 7, 9, and 11 were designated product use days while Days −1, 2, 4, 6, 8, and 10 were designated wash-out days. The subjects were housed at the test site from the time of check-in through completion of study events on Day 11. The clinic staff monitored the subjects during the confinement period to ensure that no illicit nicotine or tobacco products were used. Study investigators were not blinded, but the subjects were as the all the e-cigarette products appeared the same. The menthol product, however, was easily discernible due to taste.

Previous studies have demonstrated that a lack of familiarity with e-cigarette products may result in low nicotine intake with their use [7, 18]. In order to allow study participants to become familiar with the e-cigarette devices, subjects were instructed on the appropriate use of the products and were required to demonstrate the appropriate use to clinic staff. Each subject was then provided with two units of the menthol and non-menthol 2.4 % nicotine products containing glycerin for at-home use prior to the start of the study and instructed to use the products each day during the 7-day lead-in period.

Two types of exposures were utilized in the study on each product use day: a controlled use period followed by an *ad lib* use period. Similar 2-stage designs have been informative in prior studies evaluating the nicotine delivery and subjective effects of e-cigarettes [5–9, 19]. Enrolled subjects were randomized into one of six product usage sequences (Table 1) with four subjects per sequence.

**Table 1:** Summary of study demographics and FTCD scores by study product sequence and overall

Trait/Test	Category/Statistic	Study product sequence						
		ABFCED (*N* = 4)	BCADFE (*N* = 4)	CDBEAF (*N* = 4)	DECFBA (*N* = 4)	EFDACB (*N* = 4)	FAEBDC (*N* = 4)	Overall (*N* = 24)
Sex	Female	2 (50 %)	2 (50 %)	2 (50 %)	2 (50 %)	2 (50 %)	2 (50 %)	12 (50 %)
	Male	2 (50 %)	2 (50 %)	2 (50 %)	2 (50 %)	2 (50 %)	2 (50 %)	12 (50 %)
Race	American Indian/Alaska Native	1 (25 %)	0 (0 %)	0 (0 %)	0 (0 %)	0 (0 %)	0 (0 %)	1 (4 %)
	Black or African American	0 (0 %)	1 (25 %)	0 (0 %)	2 (50 %)	0 (0 %)	0 (0 %)	3 (13 %)
	White	3 (75 %)	3 (75 %)	4 (100 %)	2 (50 %)	4 (100 %)	4 (100 %)	20 (83 %)
Ethnicity	Hispanic or Latino	1 (25 %)	0 (0 %)	0 (0 %)	0 (0 %)	0 (0 %)	0 (0 %)	1 (4 %)
	Not Hispanic or Latino	3 (75 %)	4 (100 %)	4 (100 %)	4 (100 %)	4 (100 %)	4 (100 %)	23 (96 %)
Age (yrs)	Mean	38.3	40.5	38.3	35.3	34.0	45.8	38.7
	SD	12.04	15.70	15.44	4.03	9.49	6.13	10.77
Height (cm)	Mean	167.25	175.75	168.75	172.75	167.00	171.75	170.54
	SD	3.403	10.243	6.131	3.775	8.206	9.811	7.331
Weight (kg)	Mean	84.1	79.2	70.8	74.7	80.0	83.9	78.8
	SD	16.52	25.89	6.15	13.13	12.93	14.14	14.91
BMI (kg/m^2^)	Mean	30.050	25.198	24.798	25.015	28.948	28.375	27.064
	SD	6.0439	5.7622	0.6854	3.8464	6.3810	4.3255	4.8512
Fagerström Test for Cigarette Dependence Score	Mean	4.0	5.3	5.0	6.0	4.0	7.8	5.3
	SD	2.16	0.5	1.41	2.16	2.16	2.63	2.18
	Median	3.5	5.0	4.5	5.5	4.5	8.5	5.0
	Minimum	2	5	4	4	1	4	1
	Maximum	7	6	7	9	6	10	10

Given the wide variability in smoking behaviors, the controlled use period of the study was intended to provide some degree of standardization of the nicotine “dose” associated with each of the study products to better understand potential influences of different vehicles, flavor characteristics and nicotine content on measured parameters. As such, the controlled use period of the study consisted of 50 puffs of the assigned e-cigarette product (5-s puffs at 30-s intervals, approximately 24.5 min of use) and smoking one conventional tobacco cigarette (30-s intervals with the subjects’ normal puff duration, approximately 4.5 min of use). Fifty e-cigarette puffs was selected as an optimal controlled “dose” as it approximated the dose of nicotine delivered by the cigarette used in the study (~0.8 mg) based on machine yields of the e-cigarettes determined previously using a standardized Canadian Intense puffing protocol [20, 21]. Thus, this controlled “dose” period was intended to reflect intensive usage of the e-cigarettes for comparison to the tobacco cigarette used in the study. During the controlled use period, subject puff counts were monitored by the clinical staff and all e-cigarettes were weighed before and after use in order determine the amount of solution consumed.

Further, evaluation under the *ad lib* use conditions provided plasma nicotine levels under uncontrolled, natural use conditions and insights into product use behaviors accompanied by subjective self-assessments of smoking urge. During *ad lib* use, subjects assigned to an e-cigarette or tobacco cigarette product were allowed to use the product as desired for the entire hour (use of the assigned e-cigarette products in an unrestricted manner or smoking as many tobacco cigarettes as they chose) and subjects were responsible for maintaining their own puff counts. All e-cigarette products were weighed before and after use. The *ad lib* use period was conducted immediately following the end of the controlled product use session. Blood samples for plasma nicotine determinations, smoking urge assessments, blood pressure, pulse rate, and exhaled CO measurements were also obtained at scheduled time points on each product use day.

### Pharmacokinetics (PK) - plasma nicotine

Blood samples for the measurement of plasma nicotine concentrations were taken by direct venipuncture at 10 min prior to, and at 5, 10, 15, 20, 25, 30, 45, 60, 75, and 90 min following the start of the controlled product usage on Days 1, 3, 5, 7, 9, and 11. Plasma nicotine was analyzed by LC-MS/MS using validated analytical methods with appropriate quality controls in accordance with applicable FDA Good Laboratory Practice regulations (Title 21 CFR Part 58). The limit of quantification for nicotine was 0.200 ng/mL.

The following PK parameters were calculated:

- **C**_**max0–30**_ - maximum observed concentration from time zero to 30 min.
- **AUC**_**0–30**_ - area under the concentration-time curve from time zero to 30 min.
- **t**_**max0–30**_ - time of the maximum concentration from time zero to 30 min.
- **AUC**_**30–90**_ - area under the concentration-time curve from 30 to 90 min.
- **C**_**90**_ - maximum observed concentration at 90 min.

### Pharmacodynamics – smoking urge

Smoking urge was assessed using a simple and subjective 100 mm visual analog scale (VAS). Various forms of VAS have been used in e-cigarette studies as a tool for obtaining various subjective effects data associated with e-cigarette use and measuring nicotine and smoking abstinence symptom suppression [6–10]. Participants were asked to rate “how strong is your urge to smoke right now?” by placing a line through a 100 mm line where far left indicated: ‘not at all’ and far right indicated: ‘extremely’. Assessments occurred within 1 min prior to the −10 (pre-product use), 5, 15, 25, 30, 60, and 90-min PK blood draws. The smoking urge change-from-baseline was calculated as the difference between the pre-use and post-use smoking urge result for each tested product.

The following pharmacodynamic (PD) parameters were calculated:

- **E**_**max0–30**_ - Maximum smoking urge change-from-baseline from time zero to 30 min.
- **E**_**maxreduction0–30**_ - Maximum smoking urge reduction from baseline from time zero to 30 min.
- **AUEC**_**0–30**_ - Area under the effect curve (smoking urge change-from-baseline) from time zero to 30 min.
- **t**_**Emax0–30**_ - Time of the maximum smoking urge change-from-baseline from time zero to 30 min.
- **AUEC**_**30–90**_ - Area under the effect curve (smoking urge change-from-baseline) from 30 to 90 min.
- **E**_**90**_ - Observed smoking urge change-from-baseline at 90 min.

### Tolerability and Adverse Events (AEs)

Tolerability evaluations included assessments of AEs, vital signs and concomitant medications. AEs reported by the subjects or observed by the clinic staff were assessed for severity (mild, moderate, or severe), as serious or not serious, and relationship to the study products (unrelated, unlikely, possible, probable, or definite) by the Principal Investigator. A study product use-emergent AE was defined as an AE that started or intensified at the time of or after study product usage. An AE that occurred during the washout period between study products was considered study product use-emergent for the last study product given. All reported AEs were coded with Medical Dictionary for Regulatory Activities (MedDRA®), Version 17.0. AEs were recorded by frequency by study product and the number of subjects experiencing product use-emergent AEs.

### Data analyses

Non-compartmental PK and smoking urge parameters were calculated using Phoenix® WinNonlin® Version 6.3 (Certara, Princeton, NJ) from the individual nicotine concentration and smoking urge change-from-baseline data. All statistical summarizations and comparisons were calculated using SAS® Version 9.3 (SAS, Cary, NC). Analyses of variance were performed on the plasma nicotine AUC_0–30_, AUC_30–90_, Cmax_0–30_, and C_90_ parameters, and a Wilcoxon Signed Rank Test was used for the t_max0–30_ parameter to assess differences between the e-cigarettes and the tobacco cigarette. Usual brand cigarette flavor (menthol or non-menthol) was included in each of the analyses as a covariate to account for any impact that flavor preference might have had on the results. Repeated measures ANOVA with simple contrasts were used to compare the maximum observed concentrations of plasma nicotine within the first 30 min and at 90 min (Cmax_0–30_ and C_90_) to the pre-product use concentration. The smoking urge parameters AUEC_0–30_, AUEC_30–90_, E_max0–30_, E_90_, and t_max0–30_ were compared using methods similar to those used in the PK analyses. Differences were considered statistically significant at an alpha level of 5 %.

## Results and discussion

### Participant characteristics

Of the 24 subjects who were enrolled into the testing phase of the study and randomized to study product sequences, 23 subjects completed the study and one subject withdrew due to a family emergency. A summary of the subjects’ demographics together with the results of the Fagerström Test for Cigarette Dependence (FTCD) scores [22, 23] for all study participants by study product sequence and overall is presented in Table 1.

### Product use

The mean pre-to-post use product weight differences for the e-cigarettes and the mean puffs taken from all products during each period of the study are provided in Table 2. During controlled product administration, all subjects were to take the same number of puffs (50) from the e-cigarette products with a defined inhalation pattern (5-s puffs every 30 s) while monitored by the clinical staff in an attempt to standardize nicotine intake. The resulting mean weight differences were comparable across study products (0.2238 - 0.2570 g), with the highest and lowest estimated nicotine delivery from the 1.6 % nicotine products. During the *ad lib* product use period, subjects were free to puff as often as they chose with no limitation on puff duration. As a result, the number of puffs from each of the e-cigarette products varied widely across subjects during the *ad lib* use period. However, the mean puff counts were fairly comparable across products, averaging between 49.5 and 60.3, with the highest and lowest mean puff counts again noted with the 1.6 % nicotine products. Product weight differences followed the same pattern. Hence, there was no clear indication in either period that subjects compensated during use of the lower nicotine-containing products by taking more or deeper puffs in an attempt to self-administer more nicotine.

**Table 2:** Product use summary

	Controlled Use Period		*Ad Lib* Use Period	
E-Cigarette	Puffs	Pre-to-Post Use Weight Difference (g)	Puffs	Pre-to-Post Use Weight Difference (g)
2.4 % nic + Gly	50.0 (50–51)	0.2251 ± 0.0408	52.3 (5–128)	0.1467 ± 0.0843
*N* = 23				
2.4 % nic + Gly/PG	50.0 (50–51)	0.2349 ± 0.0633	55.4 (4–136)	0.1445 ± 0.0880
*N* = 23				
2.4 % nic + Gly + menthol	50.0 (50–51)	0.2421 ± 0.0477	58.0 (8–140)	0.1604 ± 0.0827
*N* = 23				
1.6 % nic + Gly	50.0 (50–51)	0.2238 ± 0.0399	60.3 (8–112)	0.1738 ± 0.0937
*N* = 23				
1.6 % nic + Gly/PG	50.0 (50–51)	0.2570 ± 0.0433	49.5 (3–118)	0.1194 ± 0.0675
*N* = 23				
	Puffs	Cigarettes Smoked	Puffs	Cigarettes Smoked
Tobacco Cigarette	10.5 (9–13)	1.0 (NA)	38.6 (16–103)	3.6 (2–7)
*N* = 24				

Values for puff count and cigarettes smoked are presented as mean (range). Values for the pre-to-post weight differences are presented as mean ± SD

*nic* nicotine, *Gly* glycerine, *PG* propylene glycol

The mean differences in product weights were observed to be smaller for each e-cigarette (~22 % - 54 %) following *ad lib* product use compared to the controlled product administration despite the subjects having taken similar numbers of puffs, or more, during *ad lib* product use. This was likely due to shorter puff durations during *ad lib* product use.

For the tobacco cigarettes, subjects smoked only a single cigarette during the controlled product administration, with an average of 10.5 puffs per cigarette taken. During the *ad lib* phase subjects smoked an average of 3.6 cigarettes, with approximately the same number of puffs taken per cigarette.

### Nicotine pharmacokinetics and blood plasma levels

**Fig. 1:**
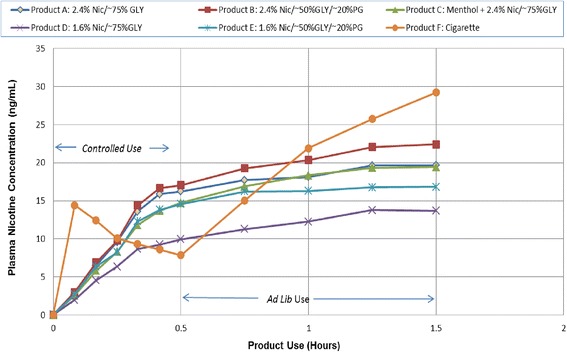
Mean Plasma Nicotine Concentration Versus Time

The mean plasma nicotine concentration-time profiles are presented in Fig. 1. Baseline nicotine concentrations were below the limit of quantification in the majority of subjects and mean baseline nicotine concentrations were comparable across study products, all representing less than half the limit of quantification of 0.200 ng/mL. During the controlled use period, mean concentrations increased peaked immediately after use of the single tobacco cigarette (*p* < 0.05 compared to baseline) and then steadily decreased until reaching a minimum at the end of the period. For all e-cigarettes, nicotine plasma absorption was slower, with mean concentrations steadily increasing with continued product use and peaking at the end of the period. Usage of the study e-cigarettes resulted in statistically significant (*p* < 0.05) increases from baseline in nicotine concentration as soon as 5 min following the start of product use with the exception of the 1.6 % nicotine product with glycerin, which also reached statistical significance after 10 min of use. Plasma nicotine concentrations steadily increased during the one-hour *ad lib* use of the tobacco cigarettes, whereas *ad lib* use of the e-cigarettes resulted in plasma nicotine concentrations that increased during the first 45 min and then plateaued during the last 15 min.

The summary of nicotine plasma PK parameters is presented in Table 3. The controlled e-cigarette puffing regimen provided peak (C_max30_) and overall (AUC_0-30_) nicotine exposures comparable to the tobacco cigarette for the two non-menthol 2.4 % nicotine products and the 1.6 % nicotine product containing glycerin and PG (all differences within 15 % of the tobacco cigarette). However, use of 1.6 % nicotine product containing only glycerin provided significantly lower peak (35 %) and overall (39 %) exposures despite having the highest mean puff counts of any of the e-cigarette products. Use of the menthol product (2.4 % nicotine with glycerin) also yielded a significantly lower overall exposure (16 %) compared to the tobacco cigarette, but a comparable peak concentration. Not surprisingly, the use pattern of the e-cigarette during the controlled use period resulted in a significantly longer time to peak nicotine concentration (T_max0–30_) than for the single tobacco cigarette.

**Table 3:** Summary of plasma nicotine pharmacokinetic parameters and statistical comparisons to the tobacco cigarette

	E-Cigarettes					
Pharmacokinetic Parameters	2.4 % nic + Gly	2.4 % nic + Gly/PG	2.4 % nic + Gly + menthol	1.6 % nic + Gly	1.6 % nic + Gly/PG	Tobacco Cigarette
	*N* = 23	*N* = 23	*N* = 23	*N* = 23	*N* = 23	*N* = 24
C_max0–30_ (ng/mL)	17.4 ± 5.97	18.1 ± 6.47	15.3 ± 5.16	10.3 ± 3.70*	15.1 ± 4.61	15.8 ± 8.64
*p*-value	0.1242	0.0508	0.8858	0.0002	0.7776	
T_max0–30_ (hr)	0.50 (0.33, 0.55)	0.50 (0.33, 0.52)	0.50 (0.33, 0.52)	0.50 (0.33, 0.61)	0.50 (0.33, 0.62)	0.09 (0.08, 0.42)
*p*-value	<0.0001	<0.0001	<0.0001	<0.0001	<0.0001	
AUC_0–30_ (ng*hr/mL)	4.7 ± 1.84	4.9 ± 1.75	4.1 ± 1.63	3.0 ± 1.18	4.3 ± 1.39	4.9 ± 1.79
*p*-value	0.7636	0.8443	0.0406	<0.0001	0.0928	
AUC_30–90_ (ng*hr/mL)	22.4 ± 5.61	24.6 ± 7.99	21.6 ± 5.08	14.7 ± 5.15	19.8 ± 4.72	22.1 ± 5.98
*p*-value	0.8463	0.0602	0.6319	<0.0001	0.0756	
C_90_ (ng/mL)	19.7 ± 5.72	22.4 ± 7.65	19.4 ± 5.80	13.7 ± 5.98	16.8 ± 4.44	29.2 ± 10.86
*p*-value	<0.0001	<0.0001	<0.0001	<0.0001	<0.0001	

T_max0–30_ is presented as median (minimum, maximum), all other values are presented as arithmetic mean ± SD. Statistical significance is based on the differences in least squares means between groups. *P*-values represent the comparison to the tobacco cigarette

*nic* nicotine, *Gly* glycerine, *PG* propylene glycol

During the *ad lib* use period, overall nicotine exposure (AUC_30–90_) was comparable (differences within 11 %) between the tobacco cigarette and all e-cigarette products with the exception of the 1.6 % nicotine product with only glycerin, which provided a 33 % lower overall exposure. However, the nicotine concentration at the end of the *ad lib* use period was significantly higher following use of the tobacco cigarette compared to the e-cigarettes, with concentrations ranging from 23 % to 53 % lower after 60 min of *ad lib* use.

Overall, the exposure parameters among the e-cigarette products tended to be higher for the 2.4 % nicotine products, higher for the products containing both glycerin and PG, and lower for the product containing menthol compared to the other 2.4 % nicotine products.

### Pharmacodynamics: effects on urge to smoke

**Fig. 2:**
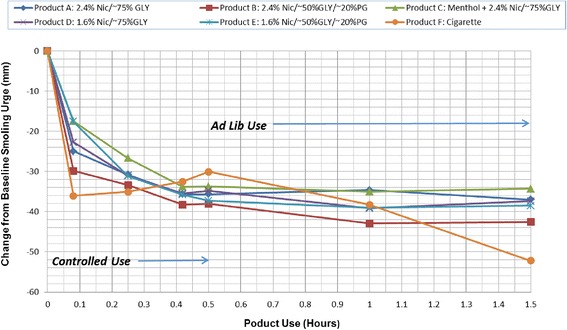
Mean Smoking Urge Change from Baseline versus Time (Note: More Negative Values Indicate a Stronger Urge Reduction)

The mean smoking urge change from baseline-time profiles are presented in Fig. 2. Mean baseline smoking urge values across all test products were comparable, with the mean visual assessment scale (VAS) responses ranging from 62 to 68 out of 100 (mm).

An immediate statistically significant (*p* < 0.001) reduction in smoking urge was observed at 5 min following the start of controlled use of each study product. Following use of a single tobacco cigarette, the maximum reduction in smoking urge was observed at 5 min, approximately corresponding to the end of use of that study product, and then increased during the remainder of the controlled use period. Continuous use of the e-cigarette products throughout the controlled use period resulted in a maximal reduction in smoking urge also approximately corresponding to the end of product use.

During the *ad lib* use period, use of the tobacco cigarette resulted in a steady decrease in smoking urge, peaking at the final time point, while the response with the e-cigarettes peaked 30 min into *ad lib* use and stabilized during the final 30 min.

Table 4 summarizes the change in smoking urge PD parameters. No statistically significant differences were found in the maximal smoking urge reduction parameters E_max0–30_ and E_maxreduction0–30_ (all differences within 11 %) or the overall smoking urge reduction parameter AUEC_0-30_ (all differences within 21 %) between the use of a single tobacco cigarette and each of the e-cigarettes during the controlled use period. The mean time to the maximal reduction in smoking urge was shorter for the tobacco cigarette (15 min) than each of the e-cigarettes (25 – 30 min), but only significantly so for both of the 1.6 % nicotine products and the 2.4 % nicotine product with menthol.

**Table 4:** Summary of change in smoking urge parameters and statistical comparisons to the tobacco cigarette

	E-Cigarettes					
Smoking Urge Parameters	2.4 % nic + Gly	2.4 % nic + Gly/PG	2.4 % nic + Gly + menthol	1.6 % nic + Gly	1.6 % nic + Gly/PG	Tobacco Cigarette
	*N* = 23	*N* = 23	*N* = 23	*N* = 23	*N* = 23	*N* = 24
E_max0–30_ (mm)	−41.9 ± 26.56	−44.7 ± 26.98	−37.0 ± 30.25	−40.2 ± 32.47	−40.7 ± 24.25	−41.5 ± 27.43
*p*-value	0.9285	0.6427	0.3023	0.6972	0.7450	
E_maxreduction0–30_ (mm)	−42.2 ± 26.01	−44.7 ± 26.98	−39.1 ± 25.86	−41.4 ± 30.40	−40.7 ± 24.25	−41.7 ± 27.08
*p*-value	0.9489	0.6505	0.5056	0.8480	0.7079	
AUEC_0–30_ (hr*mm)	−14.1 ± 10.56	−15.5 ± 10.39	−12.2 ± 10.75	−13.9 ± 12.09	−13.3 ± 8.29	−15.4 ± 12.67
*p*-value	0.4054	0.8912	0.0882	0.3590	0.2225	
TE_max0–30_ (hr)	0.41 (0.07, 0.50)	0.41 (0.07, 0.50)	0.41 (0.08, 0.50)	0.42 (0.08, 0.50)	0.49 (0.24, 0.50)	0.25 (0.07, 0.51)
*p*-value	0.1764	0.0948	0.0014	0.0012	0.0009	
AUEC_30–90_ (hr*mm)	−44.3 ± 35.39	−50.9 ± 33.64	−42.7 ± 38.24	−46.2 ± 40.23	−47.7 ± 34.16	−46.9 ± 30.14
*p*-value	0.6679	0.5275	0.5046	0.8919	0.9395	
E_90_ (mm)	−37.0 ± 30.69	−42.5 ± 29.85	−34.3 ± 34.03	−37.4 ± 35.08	−38.5 ± 30.12	−52.2 ± 26.33
*p*-value	0.0060	0.0921	0.0010	0.0066	0.0122	

TE_max0–30_ is presented as median (minimum, maximum), all other values are presented as arithmetic mean ± SD. Statistical significance is based on the differences in least squares means between groups. *P*-values represent the comparison to the tobacco cigarette

*nic* nicotine, *Gly* glycerine, *PG* propylene glycol

During the *ad lib* use period, the overall smoking urge reduction (AUEC_30-90_) achieved with use of the e-cigarettes were comparable to that of the tobacco cigarette, with all differences less than 10 %. However, at the end of the *ad lib* period, use of the tobacco cigarette resulted in a 19 % to 34 % greater relief of smoking urge (E_90_) when compared to the e-cigarettes, with statistically significant differences for all except the 2.4 % nicotine product containing both glycerin and PG.

The smoking urge reduction appeared comparable among the e-cigarette products with the exception of the 2.4 % nicotine product with menthol which tended to provide a somewhat lower level of relief compared to the other four products.

### Tolerability and reported adverse events

During the course of the study, a total of 38 subjects were exposed to one or more of the study products. There were no serious adverse events reported and no subjects were discontinued due to AEs. Mild product-use-emergent AEs were reported by 18 of 38 subjects provided a study product (including the lead-in period). The number of subjects reporting AEs was similar among products, ranging from 3 to 10 subjects each, with slightly fewer subjects experiencing AEs following use of the menthol-flavored product. The most frequent AE was cough, reported 20 times by 11 subjects (more commonly with use of an e-cigarette product than the tobacco cigarette), followed by throat irritation (8 reports by 5 subjects) and headache (6 reports by 5 subjects), and dizziness (5 reports by 4 subjects). All AEs resolved without sequelae.

The observed acute effects of the study products on blood pressure, heart rate and CO levels were previously reported by the authors under a separate publication [24] where it was noted that heart rate and systolic and diastolic blood pressure were significantly elevated after the use of the tobacco cigarette, but the elevation was less after use of most of the e-cigarettes. Furthermore, it was also observed that the use of the e-cigarettes produced no increase the exhaled CO levels, whereas the cigarette significantly increased the exhaled CO more than eight (8) times above the baseline.

## Conclusions

The key objectives of this study were to examine the nicotine blood plasma levels and smoking urge impacts of various formulations of one brand of e-cigarette for comparison to a conventional tobacco cigarette, and to assess the tolerability and adverse events associated with the study products following short-term use under intensive and naturalistic use conditions. The study design and results are not intended to support the potential for e-cigarettes as harm-reduction products.

While not all puffing parameters can be controlled across all subjects, a product use that includes the same number of puffs, puff duration, and puff interval as utilized in this study allows for standardization of nicotine intake to the extent possible in order to accurately characterize uptake and reduction in smoking urge. The small difference in pre-to-post use weight differences across the e-cigarette products supports this. When compared to a tobacco cigarette, the nicotine PK following a controlled, 50-puff use of the e-cigarettes was characterized by slower absorption than from a single tobacco cigarette, but comparable maximal and overall nicotine exposures for all but the 1.6 % nicotine product with glycerin. Analysis of the mean maximum plasma concentrations attained during the controlled use period showed that the non-menthol e-cigarettes containing 2.4 % nicotine exceeded that of the tobacco cigarette (15.8 ng/mL at approximately 5 min) with approximately 25 min of use (17.8 to 18.1 ng/mL). This observation is most likely attributable to the rather intensive e-cigarette puffing regimen (one puff every 30 s) imposed during the controlled use interval when compared to the single tobacco cigarette regimen (average 10.5 puffs total). However, it also suggests that under an intensive puffing scenario, e-cigarettes are capable of delivering similar amounts of nicotine as a tobacco cigarette.

The continuous rise in nicotine concentrations achieved with use of the e-cigarettes during the controlled use period appeared to be matched by a consistent decrease in smoking urge, though at the end of the 30-min controlled use period the urge reduction was comparable across those products. Smoking a single cigarette during the controlled use period produced a predictable nicotine concentration-time curve, with a rapid peak nicotine concentration followed by a gradual elimination. The rise and fall of nicotine concentration was matched by the smoking urge response, with a rapid decrease in urge followed by a gradual return toward baseline. While the time to maximum urge reductions was significantly shorter for the tobacco cigarette during the controlled use period compared to each of the e-cigarettes, there were no significant differences in the maximal or overall urge reduction achieved between the two types of products.

*Ad lib* use of the study products can provide insight into use patterns and nicotine concentrations that will allow consumers to satisfy their smoking urge and provide an overall satisfying product use experience. As should be expected, individual use of the products as measured by puff counts varied widely and followed the amount of nicotine solution that was consumed during use as measured by pre-to-post use product weight difference. Use of the e-cigarettes during the *ad lib* use period resulted in a relatively small increase in nicotine concentration during the first 45 min, followed by a plateau during the last 15 min. In contrast, use of the cigarette during the *ad lib* use period resulted in a continuous increase in nicotine concentration and continued reduction in smoking urge through the final time point.

Following *ad lib* use of all products for 60 min, the tobacco cigarette yielded a significantly higher nicotine concentration (C_90_) compared to each of the e-cigarettes. The higher nicotine concentration coincided with a greater reduction in smoking urge at the end of the *ad lib* use period (E_90_) for the tobacco cigarette compared to all e-cigarettes, and significantly greater than all except the 2.4 % nicotine product containing glycerin and PG. However, there was no overall impact on urge as assessed by AUEC_30–90_. This was likely due to the relatively short duration of the *ad lib* use. Indeed, as the smoking urge response appeared to have reached a plateau with the e-cigarettes and continued to increase with the cigarette following 60 min of *ad lib* use, a longer evaluation period may have resulted in a difference in the AUEC parameter as well.

Among the e-cigarettes, use of the e-cigarettes containing 1.6 % nicotine resulted in lower nicotine exposure compared to the e-cigarettes containing 2.4 % nicotine. However, this did not translate into significant differences in the smoking urge response. Further, while the suppression of urge to smoke appeared comparable between the 1.6 % and the 2.4 % test formulations in the present study, evaluation of the mean puff counts and the amount of solution consumed did not suggest that the subjects compensated by puffing on the lower-nicotine products consciously or subconsciously more so than the higher-nicotine products to achieve a similar level of satisfaction. In addition, higher nicotine content, the presence of PG in the vehicle, and the absence of menthol in the e-cigarettes were found to increase plasma nicotine levels during both controlled and *ad lib* use. Such factors, however, did not appear to significantly affect smoking urge. However, consistent with prior research documenting the prominent role of conditioned behavior and nicotine addiction, in addition to the concentration of nicotine, the respiratory tract sensory cues and manipulation of smoking materials that are associated with smoking, and mimicked by e-cigarettes, may have had a role in relieving smoking urges [25]. Thus, the present findings further suggest that some of the substantial sensory and behavioral aspects of smoking that are mirrored by e-cigarettes, may be essential elements in the reduction of craving or abstinence symptoms. To the extent that e-cigarettes may provide some measure of reduction in smoking urges, they may serve as cigarette substitutes for smokers who would otherwise seek to smoke a conventional tobacco cigarette.

Despite the aggressive puff regime employed in the controlled use period, there were no SAEs in this study and no subjects were discontinued due to AEs. The incidence of AEs was similar among products, with slightly fewer subjects experiencing AEs following use of the menthol-flavored product. The most frequently reported minor AEs were cough, followed by throat irritation, headache, and dizziness. These findings are consistent with other research studies of longer duration that have reported that use of e-cigarettes by adult smokers is well-tolerated as compared to tobacco cigarette use [2, 26].

This study was not without limitations. It was performed at a single site with a small number of subjects, which might lead to the conclusion that the data is not generalizable to a broader population. However, many initial PK evaluations of tobacco and pharmaceutical products are performed using a similar approach. Further, the comparison to the e-cigarettes was made based on a single product use and a short-term *ad lib* product use to a single brand of tobacco cigarette. Indeed, the trend we noted at the end of the *ad lib* use period did show that nicotine concentrations appeared to be continuing to rise and smoking urge appeared to be continuing to decrease. Hence, a longer term comparison in future studies would be beneficial. Finally, while the product use data did not provide a clear indication that subjects compensated by using the products with less nicotine more intensively, use of topography measurements providing insight into puff volume and inhalation rate coupled with product evaluation questionnaires in future studies might lend additional information regarding product use behaviors leading to PK and smoking urge responses.

Overall, the findings of this study indicate that nicotine uptake from short-term use of e-cigarettes containing 1.6 % and 2.4 % nicotine are significant, but lower than those of tobacco cigarettes, yet the reduction in urge-to-smoke or craving symptoms are broadly comparable. Moreover, it was also found that the short-term use of e-cigarettes by adult smokers is well-tolerated as compared to tobacco cigarette use.

## Abbreviations

*ad lib*: *Ad lib*itum
AE: Adverse event
AUC: Area under the concentration-time curve
AUEC: Area under the effect curve
CO: Carbon monoxide
CRO: Contract Research Organization
e-Cigarettes: Electronic cigarettes
FTCD: Fagerström test for cigarette dependence
Kg: Kilogram
LC-MS/MS: Liquid chromatography-tandem mass spectrometry
mg: Milligram
mm: Millimeter
mL: Milliliter
ng: Nanogram
PD: Pharmacodynamics
PK: Pharmacokinetic
PG: Propylene glycol
ppm: Parts per million
USP: United States Pharmacopeia
VAS: Visual analog scale
